# SARS-CoV-2 infection and multi-organ system damage: A review

**DOI:** 10.17305/bjbms.2022.7762

**Published:** 2023-01-06

**Authors:** Ali A Rabaan, Samira Smajlović, Huseyin Tombuloglu, Sabahudin Ćordić, Azra Hajdarević, Nudžejma Kudić, Abbas Al Mutai, Safaa A Turkistani, Shamsah H Al-Ahmed, Nisreen A Al-Zaki, Mona J Al Marshood, Amal H Alfaraj, Saad Alhumaid, Ebtesam Al-Suhaimi

**Affiliations:** 1 Molecular Diagnostic Laboratory, Johns Hopkins Aramco Healthcare, Dhahran, Saudi Arabia; 2 College of Medicine, Alfaisal University, Riyadh, Saudi Arabia; 3 Department of Public Health and Nutrition, The University of Haripur, Haripur, Pakistan; 4 Laboratory Diagnostics Institute Dr. Dedić, Bihać, Bosnia and Herzegovina; 5 Department of Genetics Research, Institute for Research and Medical Consultations (IRMC), Imam Abdulrahman Bin Faisal University, Dammam, Saudi Arabia; 6 Cantonal Hospital “Dr. Irfan Ljubijankić”, Microbiological Laboratory, Bihać, Bosnia and Herzegovina; 7 International Burch University, Faculty of Engineering and Natural Sciences, Department of Genetics and Bioengineering, Ilidža, Bosnia and Herzegovina; 8 University of Sarajevo, Faculty of Agriculture and Food Science, Sarajevo, Bosnia and Herzegovina; 9 Research Center, Almoosa Specialist Hospital, Al Mubarraz, Saudi Arabia; 10 College of Nursing, Princess Norah Bint Abdulrahman University, Riyadh, Saudi Arabia; 11 School of Nursing, Wollongong University, Wollongong, NSW, Australia; 12 Nursing Department, Prince Sultan Military College of Health Sciences, Dammam, Saudi Arabia; 13 Fakeeh College for Medical Science, Jeddah, Saudi Arabia; 14 Specialty Pediatric Medicine, Qatif Central Hospital, Qatif, Saudi Arabia; 15 Pediatric Department, Abqaiq General Hospital, First Eastern Health Cluster, Abqaiq, Saudi Arabia; 16 Administration of Pharmaceutical Care, Al-Ahsa Health Cluster, Ministry of Health, Al-Ahsa, Saudi Arabia; 17 Biology Department, College of Science and Institute for Research and Medical Consultations (IRMC), Imam Abdulrahman Bin Faisal University, Dammam, Saudi Arabia

**Keywords:** SARS-CoV-2, angiotensin-converting enzyme 2 (ACE2), COVID-19, cytokine storm, multi-organ failure

## Abstract

The SARS-CoV-2 infection causes COVID-19, which has affected approximately six hundred million people globally as of 8 2022. Organs and cells harboring angiotensin-converting enzyme 2 (ACE2) surface receptors are the primary targets of the virus. However, once it enters the body through the respiratory system, the virus can spread hematogenously to infect other body organs. Therefore, COVID-19 affects many organs, causing severe and long-term complications, even after the disease has ended, thus worsening the quality of life. Although it is known that the respiratory system is most affected by the SARS-CoV-2 infection, many organs/systems are affected in the short and long term. Since the COVID-19 disease simultaneously affects many organs, redesigning diagnostic and therapy policies to fit the damaged organs is strongly recommended. Even though the pathophysiology of many problems the infection causes is unknown, the frequency of COVID-19 cases rises with age and the existence of pre-existing symptoms. This study aims to update our knowledge of SARS-CoV-2 infection and multi-organ dysfunction interaction based on clinical and theoretical evidence. For this purpose, the study comprehensively elucidates the most recent studies on the effects of SARS-CoV-2 infection on multiple organs and systems, including respiratory, cardiovascular, gastrointestinal, renal, nervous, endocrine, reproductive, immune, and parts of the integumentary system. Understanding the range of atypical COVID-19 symptoms could improve disease surveillance, limit transmission, and avoid additional multi-organ-system problems.

## Introduction

The SARS-CoV-2 infection causes COVID-19 disease, as named by the International Committee on Taxonomy of Viruses and the World Health Organization on February 12, 2020. The virus first appeared in China and spread globally, causing a pandemic by infecting 591 million people, contributing to over 6.4 million deaths globally [1]. So far, the world has witnessed three waves of coronavirus, and more are expected to come. The mutations led to new variants, including the Indian Delta (B.1.617.2) and the South African Omicron (B.1.1.529). Today, however, the Omicron variant has spread worldwide with an incidence of >99%. The emergence of new mutations and variants can affect the success of existing vaccines and cause waves of new coronaviruses [2].

COVID-19 is a disease characterized to be very contagious and is spread from one person to another via respiratory droplets [3]. However, once in the body, the virus can also spread hematogenously to infect other body organs [4]. In most cases, it causes mild signs and symptoms but can also lead to a severe form of the disease. It affects the respiratory system but it also attacks the central regulating systems, such as the nervous and endocrine systems that integrally play a pivotal function in adjusting homeostasis. Dysfunction of the two systems through COVID-19 significantly influences many other organs and their functions in the body. Besides these two, the virus also causes damage to the other organ systems, such as the cardiovascular, gastrointestinal, and urinary systems. The virus interacting with the angiotensin-converting enzyme 2 (ACE2) receptors causes direct injury to the organs or worsening a pre-existing systemic disease [5]. This review focuses on the interaction between the SARSCoV2 infection and various organ systems, including respiratory, immune, endocrine, nervous, gastrointestinal, cardiovascular, renal, and reproductive, and parts of the integumentary systems.

## Data sources

We thoroughly searched the literature using the keywords “COVID-19” and “SARS-CoV-2” in the PubMed, MEDLINE, Google Scholar, and medRxiv.org databases. The literature covered the period from January 1, 2020, to August 10, 2022. For the review, a total of 280 English-language articles were chosen. The articles relating to COVID-19 and each organ system were separated. Additionally, articles have been added that may be useful for future work to expand knowledge based on the evolution of the pandemic. Finally, a separate summary was written for each organ system.

## SARS-CoV-2 and respiratory system

COVID-19 has been linked to many long-term respiratory problems, including persistent symptoms and radiologically detectable alterations, poor respiratory physiology, vascular tissues, and pulmonary fibrosis. This is related to the SARS-CoV-2 infection of the lungs and the damage it causes [6]. The virus reaches the lungs via inhalation of respiratory droplets containing the viral particles [7]. It binds to cells that express the ACE2 receptors on their surfaces, thereby causing direct damage to alveolar cells, especially the Type II cells. This further triggers increased permeability of the epithelial cell wall, leading to increased fluid infiltration and lung edema. Gaseous exchange surface is also impaired, leading to hypoventilation, hypoxia, and imbalance between ventilation and perfusion. Shunting of arterial blood to other regions of the lungs occurs, leading to ventilation–perfusion-mismatch [8]. The two most common signs of severe COVID-19 illness are acute respiratory distress syndrome (ARDS) and pneumonia.

COVID-19 individuals with ARDS have a higher risk of death and sequelae and frequently require mechanical ventilation for respiratory support [9]. Severe disease can present with high blood pressure, cardiovascular disease, and chronic pulmonary disease [10]. An overactive innate immune response contributes significantly to the development of ARDS in COVID-19 individuals. The virus stimulation of resident macrophages and lung epithelia leads to local cytokine generation and neutrophil employment in this situation. Stimulated neutrophils eject a network of antimicrobial-containing DNA-based cytoplasmic material known as neutrophil extracellular traps (NETs). Even though NETs are a defense tactic versus attacking pathogens, they may operate as a nidus for the buildup of coagulation factors and activated platelets, resulting in the formation of thrombi. The immunothrombosis can cause blood vessel blockage, resulting in ischemic damage [11].

Pneumonia is a severe respiratory illness related to COVID-19 [12]. It is clinically manifested as fever, dry cough, dyspnea, difficulty breathing, chest pain, and malaise [13]. COVID-19 and chronic obstructive pulmonary disease (COPD) have several unfavorable interrelations that might impact infection progression and clinical effects. COPD patients are more vulnerable to viral infections and the pathophysiological implications of COVID-19, such as microthrombosis, intrapulmonary shunting, and bacterial infection [14]. Low-dose chest computed tomography (CT) with a chest X-ray is used as a diagnostic tool for symptomatic hospitalized patients with severe COVID-19 infection and pneumonia [15]. In the early stages of pneumonia, CT images typically display unilateral focal ground-glass opacities and rapidly transform into bilateral multilobar ground-glass opacities with a peripheral or posterior distribution. In the later stages, these structures may develop into consolidative pulmonary opacities and mixed patterns [15–17].

The nasal cavity has a high viral load and ACE2 receptor expression. It plays a significant part in transmitting SARS-CoV-2 infection, and otolaryngologists should focus on infection prevention in clinical circumstances, including nasal disorders and illnesses. Damage to sustentacular cells expressing the ACE2 receptor is the cause of COVID-19-related olfactory dysfunction, rather than damage to olfactory sensory neurons, although further research is needed [18].

Most COVID-19 respiratory problems are treated symptomatically and with supportive care [6]. Identifying the primary processes responsible for the abnormalities in gas exchange and respiratory mechanics, as well as their variations over time, should determine the best configuration of respiratory support. Monitoring inspiratory effort in spontaneously breathing patients (non-invasive or invasive ventilation) is an essential supplementary tool for informing the decision of mechanical support and identifying treatment failure [19]. Clinical, physiological, and radiological surveillance and early referral to a chest specialist are currently advised for all COVID-19 survivors [6]. Together, antibiotics have been prescribed in both hospitalized COVID-19 patients and outpatients, especially at the early times of the pandemic [20]. Since the viral infection increases C-reactive protein (CRP) and procalcitonin (PCT) values, markers for bacterial infection, a prescription for the bacterial infection is recommended. Coinfection of COVID-19 patients with bacterial infections increases the death risk. Therefore, antibiotic treatment may help to recover hospitalized COVID-19 patients. However, recent research has found that antibiotic therapy is inefficient in decreasing the death risk in patients with mild and severe COVID-19. Conversely, it leads to a higher mortality rate in COVID-19 patients when used unnecessarily without an apparent reason suggesting bacterial infection [21]. Therefore, prescribing antibiotics in COVID-19 patients should be well elucidated to reduce the mortality rate and antibiotic resistance. As a potential marker to differentiate between viral and bacterial infections, the PCT can help prevent the unnecessary use of antibiotics [22].

## SARS-CoV-2 and cardiovascular system

Cardiac damage occurs roughly in every fourth infected patient for SARS-CoV-2 infection, whereas it develops *de novo* in almost every tenth infected [23]. Cardiac damage caused by SARS-CoV-2 infection can occur in people with no history of cardiac disease or the absence of symptoms, making it difficult to identify [24]. Sex, advanced age, and chronic comorbidities have been identified as the most critical factors linked with a poor prognosis.

SARS-CoV-2 can directly infect the cardiac myocytes via the surface ACE2 receptors expressed on the cells. ACE2 is a cardiac protective factor because it causes the formation of angiotensin I from angiotensin II. SARS-CoV-2 binds to the ACE2 receptors and alters the signaling pathway, downregulating the levels of ACE2 [25]. The SARS-CoV-2 infection has been linked to several cardiovascular disorders (CVDs) in the way listed below.

CVDs caused by COVID-19 infections can occur because of:

1. *Direct myocardial damage:* Virus entrance may disrupt in the ACE2 signaling pathways, resulting in acute myocardial injury [26]. The cardiac injury was observed in 11% of the cases [27] and was more likely to be observed in elderly patients with other comorbidities such as hypertension. They were found to have increased cardiac markers, such as PCT, CRP, and creatinine [28]. Current research reveals that, whereas hypertension is widespread in COVID-19 individuals, it does not have an independent role in SARS-CoV-2 infection and COVID-19 development [29].

Individuals with injury to the heart develop complications like a disturbance in electrolyte levels, coagulation disorders, and injury to the kidneys. This subsequently contributes to the increased case fatality. Myocardial infarction due to ischemia of cardiac myocytes was indicated by elevated levels of cardiac Troponin I [30–32]. Elevated Troponin I and cardiac arrest were also observed in patients with no previous cardiovascular disease history [33]. Elevated levels of the myocardial band of the creatine kinase indicated myocardial injury [34, 35]. This was also witnessed in patients who presented with a history of cigarette smoking [36].

Increased cardiometabolic load linked with systemic infection and hypoxia induced by severe respiratory sickness might disrupt myocardial oxygen demand-supply connection and contribute to acute myocardial damage [26]. Cardiac damage from infection and inflammation is associated with myocarditis. If the infection also compromises the lungs, the oxygen supply to the heart decreases. This causes myocyte ischemia and hence myocardial infarction [46].

Furthermore, acute thrombotic episodes are prevalent in individuals with serious COVID-19, making SARS-CoV-2 a distinct virus with an unknown pathogenesis. The illness can result in venous and arterial thrombosis, especially pulmonary embolism (PE) and microthrombi. A recent study on 417,975 COVID-19 and 345,934 influenza patients revealed that COVID-19 patients had higher venous thrombosis risk than influenza patients, but not arterial thrombosis [37]. The arterial thrombosis occurs in approximately 4% of critically ill COVID-19 patients—particularly in male, elder, and comorbid ones. Many studies have reported that limb ischemia, acute cerebral ischemia, acute mesenteric ischemia, and acute myocardial ischemia have manifested in COVID-19 patients with arterial thrombosis (reviewed in [38]).

2. *Inflammation throughout the body:* The most severe outcomes of viral infection include acute systemic inflammatory response and cytokine storms. These modifications may result in multi-organ failure. Clinical test results from critically unwell individuals demonstrate a high level of circulating cytokines [26].

SARS-CoV-2 is a new virus that can produce significant alterations in blood counts, most notably severe lymphopenia and extreme fatigue of CD8+ T cells in severe instances, potentially reducing the cellular immune response of patients [39, 47]. Decreased platelets, leukocytes, and neutrophils are associated with an indigent outcome [40]. COVID-19 causes viremia later in the disease’s course and can produce severe inflammatory responses such as cytokine storms, which may require intensive care unit (ICU) hospitalization. Virus-induced disseminated intravascular coagulation is unusual, yet it resembles sepsis-induced disseminated intravascular coagulation in certain ways [39].

Cytokine storms due to COVID-19 infection result from activation of immune pathways and massive pro-inflammatory cytokines [41]. These inflammatory markers can be used to assess illness severity, including monocyte chemoattractant protein-1, tumor necrosis factor-α, CRP, interleukin-1β, and interleukin-6 (IL-6), interferon-γ, and ferritin. Cytokine storms cause increased inflammation which leads to elevated levels of ferritin. In a study in Wuhan, China, elevated levels of IL-6 and ferritin were found to catalyze mortality in COVID-19 patients [42].

While cytokine storms produce thromboinflammation, ACE2 downregulation generates RAS imbalance, which leads to systemic thrombotic microangiopathies and ischemic stroke [43]. The risk of a cytokine storm is particularly increased in patients with cardiovascular disease. There is a greater risk of COVID-19 severity and fatality of up to 2.5 folds in patients with hypertension, especially in older patients [44]. Antihypertensive drugs that block the angiotensin receptor and ACE inhibitors result in ACE2 upregulation and cause increased COVID-19 case fatality. Because of this, patients on these medications should be monitored continuously [44]. Other contrary studies suggest that using these medications to treat COVID-19 individuals may lead to an increased survival rate for these patients [45].

3. *Arrhythmias:* COVID-19 infection is associated with arrhythmias in 44% of cases [26, 27]. Increased metabolic demand by the heart occurs during inflammation and fever. This can lead to worsening heart failure. Cardiac muscle inflammation in patients with COVID-19 causes an alteration in cardiac rhythm leading to arrhythmias. Not only do these include bradycardia and tachycardia, but they also include asystole. Patients without any pre-existing risk factors can also have myocarditis which is life-threatening if the heart is severely inflamed [48].

It is critical to stratify overall risk by considering additional comorbidities, such as diabetes, neurological problems, impairments, or pulmonary diseases. The most prevalent comorbidities among COVID-19 patients who require hospitalization are hypertension (56.6%) and diabetes (33.8%) [49]. COVID-19 patients can be treated the same way as the general population unless they develop cardiac symptoms. To assess a high-risk patient with acute COVID-19 and to aid in the early discovery of patients who require hospitalization, the electrocardiogram (ECG) must be analyzed. Biomarkers, such as NT proBNP, BNP, troponins, myoglobin, D-dimers, CRP, interleukin-2, IL-6, and ferritin levels, must be measured [50]. Imaging is essential in detecting cardiovascular injury caused by SARS-CoV-2 infection monitoring patients’ clinical improvement throughout therapy, and discovering long-term disease sequelae. The sensitive non-invasive opportunity provided by radionuclide imaging using positron emission tomography (PET) may further understand the molecular disturbances that appear in many organs and contribute to an adverse results in COVID-19. PET methods might also be used to evaluate the long-term consequences of COVID-19, particularly in individuals with persistent symptoms [51].

## SARS-CoV-2 and gastrointestinal system

Gastrointestinal (GI) symptoms, observed in several patients infected with COVID-19, can occur at any stage, either starting as an initial symptom or emerging with the progression of the disease. However, GI symptoms perform differently in different clinical periods [52]. Since the GI symptoms usually appear in the initial stages of the disease, they usually remain underdiagnosed or misdiagnosed, making it difficult to correlate it with the COVID-19 infection [53]. The time interval between the onset of illness and the appearance of GI symptoms in COVID-19 individuals is not fixed and might be used to indicate the progression of the disease. It is noticed that during the COVID-19 infection, some patients experience GI symptoms exclusively, while others develop respiratory discomforts followed by GI manifestations [54]. These manifestations in most patients include diarrhea, nausea, and vomiting, with diarrhea and loss of appetite being the most frequent and usually tied to the disease severity [55–57].

The possible molecular mechanism lying behind is associated with ACE2, one of the SARS-CoV-2 binding receptors widely present in GI epithelial cells [58, 59]. The interaction among Spike proteins found on the surface of SARS-CoV-2 and ACE2 causes damage to GI epithelial cells, resulting in the loss of function of ACE2, which further produces micro ecological imbalance, intestinal inflammation, and immune disorders [60, 61].

With varying onset and severity, GI manifestations are reported in 11.4%–61.1% of COVID-19 patients. A small percentage of patients present with an acute abdomen caused by acute pancreatitis, acute appendicitis, intestinal obstruction, bowel ischemia, hemoperitoneum, or abdominal compartment syndrome. The GI tract may be involved due to direct viral injury and/or an inflammatory immune response, resulting in malabsorption, an imbalance in intestinal secretions and gut mucosal integrity, and enteric nervous system activation. The primary focus of therapy is on supportive and symptomatic care. However, some patients may require surgical or endoscopic treatment for acute abdominal and GI bleeding [62].

Diarrhea has emerged as an early symptom of COVID-19 due to its prevalence in otherwise asymptomatic COVID-19 patients [63]. According to reports, diarrhea appears between 1 and 8 days after infection, with a mean onset of 3.3 days [64]. Diarrhea has been reported in 2%–50% of cases [65] and was found to be more common in severe diseases (moderate 69.2%, severe 100%) [66]. Han et al. [67] discovered that diarrhea was the first symptom in 20% of their patients, while the rest experienced it up to 10 days after the onset of respiratory symptoms. Furthermore, most patients experience diarrhea in the hospital because of medications or other treatments. Antibiotics, for example, can disrupt the intestinal microbiome and result in antibiotic-associated diarrhea [68].

Nausea had an abrupt onset and was an early warning of a problem in the upper digestive tract and component of the body’s epithelial defenses, implying that nausea could be the first sign of SARS-CoV-2 infection in the GI tract [69]. A higher incidence of nausea was linked to a more severe disease. Patients who present with nausea, vomiting, and diarrhea are more likely to have fever than those who only have one of the symptoms [67]. Vomiting and milk refusal were observed in COVID-19 neonates and respiratory symptoms [70].

Abdominal pain was lower than other GI symptoms but was common in patients receiving ICU care [71]. Acute pancreatitis, acute appendicitis, intestinal obstruction, small bowel ischemia, sigmoid ischemia, haemoperitoneum, haemopneumoperitoneum, or abdominal compartment syndrome were among the causes of abdominal pain in a minority of patients [62, 69].

Upper GI endoscopy was deemed a high-risk procedure due to the increased risk of SARS-CoV-2 virus aerosolization and transmission [72]. Upper GI endoscopy, nasogastric tube insertion, caustic injury, and surgery are all common causes of esophageal rupture. Transmural perforation that occurs after forceful vomiting is known as spontaneous perforation. It is also known as Boerhaave syndrome [73].

A patient with severe respiratory and GI symptoms should be diagnosed with Boerhaave syndrome as soon as possible. To prevent death from sepsis and multiple organ failure, rapid implementation of primary interventions, which include sepsis control, adequate drainage, perforation repair, and antibiotic therapy is critical [74].

In research published by Rahman et al. [75], a patient was diagnosed with Boerhaave syndrome based on his esophageal rupture, which was caused by his COVID pneumonia-related coughing and vomiting. The proper management was initiated within the first 24 h, significantly impacting the patient‘s clinical outcome. The patient survived despite Boerhaave syndrome’s rarity and resource scarcity during the COVID-19 pandemic [75].

With respect to all of the evidence and literature that has been published thus far on COVID-19, it is highly advised that all medical authorities and scientists should be prepared to diagnose and treat various COVID-19 complications and unusual presentations [75].

SARS-CoV-2 causes direct injury to the liver hepatocytes via the surface ACE2 receptors making it the second most affected organ after the lungs [8]. Liver damage in COVID-19 individuals has been associated with elevated levels of inflammatory mediators, hypoxia, and cytokine storms [76, 80]. Besides hepatocytes, ACE2 is present in cholangiocytes (60%). However, Kupffer cells, for example, have no ACE2 receptors [77–79].

The more severe form of SARS-CoV-2 infection is seen in individuals who present with underlying liver disease, including viral hepatitis [80]. Serum enzyme levels of the liver, like lactate dehydrogenase, are elevated, suggesting liver injury from immune complexes or drugs. However, antiviral drugs, such as remdesivir, lopinavir, and hydroxychloroquine, which have clinically prescribed for use in COVID-19 disease management, may cause liver damage. This may increase drug toxicity or aggravate the severity of SARS-CoV-2 infection [81].

A pathological examination of liver biopsy specimens from a COVID-19 patient revealed moderate microvesicular steatosis and mild lobular and portal activity, indicating that SARS-CoV-2 may have caused this liver damage [82].

However, little data has thoroughly examined other liver enzymes and clinical characteristics of liver failure in COVID-19 patients. Cai et al. [83] described the liver test results in COVID-19 patients. Patients with abnormal liver test results, particularly hepatocyte type or mixed type, had significantly higher risks of developing severe pneumonia when compared to patients with regular liver tests at admission. Because almost all patients had liver tests when they were admitted, abnormalities in liver tests can be used to predict disease severity [85]. Patients with elevated hepatocyte-type liver enzymes at admission or during hospitalization had a significantly higher risk of progressing to severe COVID-19 [83].

The rate of abnormal liver tests in their study was higher than previously reported, and only a small proportion had underlying liver disease, implying that liver damage in patients with coronavirus infection may be directly caused by viral infection of liver cells [84].

The effect of COVID-19 in patients with chronic liver disease is largely unknown. The data published thus far demonstrate the importance of monitoring liver function in hospitalized COVID-19 patients, not only for the potential risk of developing acute liver failure but also for stratifying patients who may benefit from early access to the ICU. Monitoring liver function tests in COVID-19 patients may help the authorities be aware that if patients’ respiratory function deteriorates, they may benefit from early transfer to the ICU before the onset of a manifest moderate-to-severe ARDS [85].

The β-cells in the pancreas express ACE2 receptors. Diabetes mellitus (DM) has been related to increased expression of ACE2 receptors on these insulin-secreting pancreas cells [86]. Individuals with pre-existent DM are particularly susceptible to developing severe COVID-19 disease. Glucose tolerance is interfered with due to reduced secretion of the insulin hormone, which plays a significant role in glucose level regulation in the body. Damage to islet cells of the pancreas may cause insulin-dependent DM [86].

Viral pancreatitis is also possible to develop [87]. Symptoms of GI infection, such as diarrhea, abdominal pain, nausea, and vomiting, may manifest as gastroenteritis, suggesting the possibility of viral transmission fecal-orally [88].

## SARS-CoV-2 and urinary system

The digestive and urinary systems and the respiratory tract are important sites of viral transmission. Because ACE2 receptors are extensively expressed in numerous organs, such as the organs of the respiratory system, digestive tract, and kidneys, they are potential targets for SARS-CoV-2 infection. Understanding the immunological mechanisms driving GI tract and kidney impairment in SARS-CoV-2 infection would lead to more effective therapy and a reduction in infected patients’ mortality and morbidity [89].

The SARS-CoV-2 infection causes renal damage through various mechanisms. The specific process is not fully understood, but experts believe that SARS-CoV-2 damages the kidneys directly and indirectly. The virus binds to the ACE2 receptors in the kidneys, enters and destroys cells, disrupts the balance of the renin–angiotensin–aldosterone system (RAAS), activates coagulation pathways, and damages the renal vascular endothelium causing direct renal cell injury. Increased cytokines and cytokine storms, sepsis, circulatory abnormalities, hypoxemia, and the use of nephrotoxic medications all contribute to the indirect route [90].

Fan et al. [91] investigated SARS-CoV-2 infection effects on the urinary system. The vas deferens, testicular, mesenchymal, and renal tubular cells express ACE2. Past histology samples from renal tissue of COVID-19 individuals suggested the existence of tubular lesions. Direct damage of these renal cells presenting with the ACE2 has been associated with the development of renal failure [91].

Cytokine storms may also cause widespread renal dysfunctions due to the development of acute kidney injury (AKI) [92]. This is usually presented in the late stage of the disease and accompanies the development of other multi-organ failures, including liver and lungs [93–95]. Ninety percent of COVID-19 patients on mechanical ventilation acquired AKI [96]. Acute kidney failure due to hypoperfusion, septic shock, rhabdomyolysis, and hypoxia, indicated by elevated serum levels of creatinine in serum samples, was obtained from patients with COVID-19 [93]. On average, 5%–23% of persons with COVID-19 have AKI symptoms, such as increased blood creatinine and urea, hematuria, proteinuria, and histological damage [91].

SARS-CoV-2 infection in chronically ill patients with chronic renal disease has increased case severity and mortality. Such patients have a higher risk of developing pneumonia and other upper respiratory tract infections. Clinically, they present with renal signs, such as hematuria, proteinuria, uremia, elevated blood urea nitrogen levels, increased serum creatinine, increased LDH enzyme levels, high creatine kinase, and D-dimer levels. A raised D-dimer was a poor prognosticator in COVID-19 patients [52]. Li et al. (2020) studied 193 COVID-19 patients. Chronic kidney disease was found in 54% of cases, 34% had severe COVID-19, and 8% had an underlying respiratory illness. A considerable number of severe cases developed complications which included septic shock (18%), ARDS (28%), acute cardiac injury (12%), and IRA (28%) [95]. Higher COVID-19 mortality was also seen in patients who have had kidney transplants [96].

## SARS-CoV-2 and nervous system

Most COVID-19 patients have moderate respiratory symptoms such as a dry cough and dyspnea, presenting with a fever. However, COVID-19 has also been linked to various neurological symptoms at the diagnosis or throughout the disease. Over 90% of individuals with COVID-19 have reported at least one subjective neurological symptom, emphasizing the relevance of the disease’s later neurological consequences. Thus, it has been noted that even after a patient has recovered, they may still have neurological and psychosocial problems [97].

**Figure 1.**
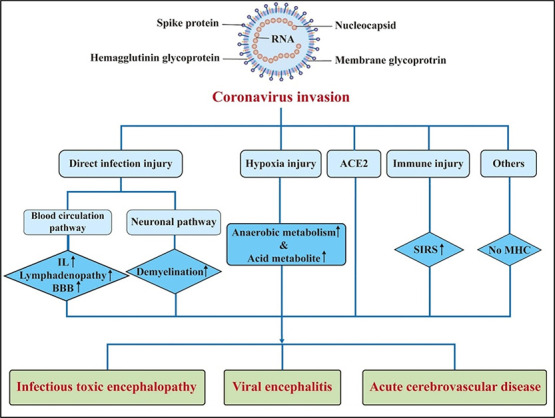
**Pathogenesis of central nervous system (CNS) injury caused by coronaviruses.** BBB: Blood brain barrier; IL: Interleukin; SIRS: Systemic inflammatory response syndrome; ACE2: Angiotensin-converting enzyme 2; MHC: Major histocompatibility complexes [108].

Studies revealed that the ACE2 receptors are expressed in the brain stem and the cerebral cortex. The SARS-CoV-2 virus can directly invade the central nervous system (CNS) via the ACE2 receptors or hematogenously [98, 99]. Invasion of the ethmoidal structures can cause viral propagation into the brain or via sensory nerve receptors found in the lungs [100]. S spike protein allows the endothelial cell receptors and the virus to interact, infect, and be transported to other brain cells, including neurons, to cause damage to the endothelium [98]. Hypoxia induced by the SARS-CoV-2 infection on the respiratory system can also cause indirect damage to the brain tissues (Figure 1). Brain hypoxia causes ischemia which can manifest as stroke [100].

Neurological manifestations can be grouped into two broad classifications: those manifesting on the CNS level and those manifesting at the level of the peripheral nervous system (PNS). The first classification includes an altered level of consciousness which include symptoms, such as: delirium, headache, dizziness, global confusion, inability to rouse, encephalopathy, syncope, convulsions, movement coordination abnormalities, cerebrovascular events like stroke, and viral encephalitis. The second classification includes neuropathic manifestations involving: olfactory, facial, cranial, and glossopharyngeal nerves. A meta-analysis revealed that cranial nerves VII, VI, and III are the most frequently influenced [101]. These can cause changes in the senses of smell and taste because of the effect on the taste receptors [102, 103]. It is important to mention that infections of the cranial nerves can result in sudden sensorineural hearing loss (SSNHL), defined as the sudden onset of sensorineural hearing loss (SNHL) with at least three consecutive frequency losses of 30 dB occurring suddenly or within three days. The occurrence of SSNHL associated with viral infections has been linked to three mechanisms:

- Neuritis caused by viral involvement of the cochlear nerves
- Cochleitis caused by viral involvement of the cochlea and perilymphatic tissues
- The stress response is generated by the cross-reaction of inner ear antigens [104–106].

The most frequent generalized neurological symptoms seen in COVID-19 individuals are headaches, confusion, and dizziness [107–109]. A few studies have found that headache is the most prevalent non-specific neurological symptom. However, headache could be mistaken for having a random relationship with COVID-19. Nevertheless, there is unambiguous evidence that COVID-19 patients who had never had a recurrent headache suddenly get a severe headache daily because of SARS-CoV-2 infection [107, 110].

Some case reports describe COVID-19 individuals who unexpectedly develop lightheadedness and a dry throat, without any additional symptoms such as fever, cough, or headache. Even though lightheadedness is irrelevant to COVID-19 seemingly, doctors must be wary of this neurological sign since COVID-19 could trigger it, mainly if there are no other symptoms [107].

A more narrow classification of neurological expressions described in the COVID-19 era includes five major categories:

1. Encephalopathy with delirium/psychosis, but no MRI or CSF abnormalities.
2. Inflammatory CNS syndromes such as encephalitis and acute disseminated encephalomyelitis (ADEM).
3. Ischemic strokes.
4. Peripheral neurological disorders such as GBS and brachial plexopathy.
5. Miscellaneous CNV disorders [111].

Severe and prolonged COVID-19 disease may initiate the onset of another neurological disease or may worsen a pre-existing one [111]. Coronaviruses have been found in patients with clinically proven optic neuritis, Parkinson’s disease, and multiple sclerosis [112–115]. Elderly individuals with severe SARS-CoV-2 infection express encephalopathy, while viral encephalitis due to SARS-CoV-2 was found in a cerebrospinal fluid (CSF) sample of an individual in Wuhan, China [116]. CSF evaluation of individuals with proven COVID-19 infection showed variation. Patients with encephalopathy primarily had increased levels of total CSF proteins, which may indicate injury to the axons. Infection by the SARS-CoV-2 virus might trigger immune cells to infiltrate the CNS, resulting in the subsequent destruction of the meninges, axons, and brain tissue [116–118]. Additionally, studies have shown an association between coronaviruses, including SARS-CoV-2, MERS-CoV, and SARS-CoV-1, infection, and the development of CNS and PNS demyelination [119, 120].

Critical illness-associated cerebral microbleeds (CIAMs) have been reported in patients with severe COVID-19 [121]. However, the underlying pathophysiological mechanisms causing diffuse microbleeds are not precise. Some hypotheses have been proposed regarding the potential role of hypoxemia, microangiopathy, or coagulopathy [122].

Some case reports showed that spinal cord dysfunction appears upon SARS-CoV-2 infection [123]. This could be related to the infection of endothelial cells and invading the virus to the spinal cord via the hematogenous route. The virus also crosses the blood–brain barrier (BBB) since it uses the inflammatory cells as a Trojan horse [124, 125]. It has been reported that spinal cord ischemia occurred after SARS-COV-2 infection due to occlusion of the Adamkiewicz artery in the thoracolumbar region. Aortic disease, thoracolumbar surgery, sepsis, hypotension, and thromboembolic disorders are the potential factors that causes this infraction [125, 126]. In addition to these factors, cytokine storms are also associated with hypercoagulation and may lead to spinal cord ischemia.

ADEM is an uncommon inflammatory CNS condition that primarily affects children. Several studies have found an increase in the prevalence of ADEM following the SARS-CoV-2 pandemics globally. ADEM cases have been associated with SARS-CoV-2 infections in several recent case reports. This implies that there might be a connection between ADEM and SARS-CoV-2 infections [127].

Some authors recognize delirium as a COVID-19 symptom [128], claiming that the existence of comorbidities discovered during the SARS-CoV-2 infection might hasten the emergence of acute delirium. Mental status alterations should be included in the list of testing requirements, regardless of the origin of delirium, since delirium might be the sole presenting symptom [107, 108].

Epilepsy from hypoxia, electrolyte imbalances, and brain death has also been associated with COVID-19 infections [129]. Inflammation of the CNS is associated with the activation of the innate immune response. This is due to viral replication and high production of pro-inflammatory cytokines that can reduce the thresholds for convulsions and promote epileptogenesis [130, 131]. The current research implies that COVID-19 can diminish the seizure threshold in people with a seizure problem due to an increase in case reports of COVID-19 individuals experiencing seizures. COVID-19 not only worsens seizure control in individuals with previously well-controlled seizures, but can also cause new-onset seizures in patients who have never had a seizure before [132].

Pro-inflammatory cytokines in animal models have been demonstrated to raise the predisposition of seizure through various mechanisms, which involve the hyperexcitability of neurons and alterations of the neuronal synapses. This neuronal inflammation and excitability outcomes were already widely reviewed in 2013 [131]. Convulsions can also be secondary to ischemic or hemorrhagic stroke [133].

Moreover, cerebrovascular diseases like ischemic or hemorrhagic stroke were more likely to appear in COVID-19 individuals with advanced age. The greater risk for hemorrhagic and ischemic stroke is posed by increased age, heart failure, hyperlipidemia, smoking, atrial fibrillation, high blood pressure, pulmonary compromise, diabetes, and previous cerebrovascular events [134, 135].

Guillain–Barré syndrome (GBS) is a rare neurological disorder in which the immune system attacks a section of the PNS by mistake (the network of nerves that extends beyond the brain and spinal cord). Acute inflammatory demyelinating polyneuropathy (AIDP) is another name for GBS, which can occur after a respiratory or GI illness. Individuals suffering from GBS may exhibit various neurological symptoms such as inflammatory polyradiculoneuropathy linked with various infections. It has been seen several times that lower limb weakness and paresthesia can progress to generalized tetraparesis or tetraplegia over a few days [108, 110, 136, 137]. SARS-CoV-2 infection may worsen pre-existing GBS [138]. In addition to GBS, Miller–Fisher syndrome (MFS), a variant of this disease, can acquire upon the SARS-CoV-2 infection. In addition to the GBS symptoms, it is called MFS when ophthalmoparesis is associated with predominantly lowerlimb involvement [139]. The MFS leads to abnormal muscle coordination, paralysis of the eye muscles, and absence of tendon reflexes, which points out the neuroinvasive capacity of coronavirus.

Psychiatric symptoms have also been documented in COVID-19 individuals. There are sequelae affecting the human body‘s neurological, cognitive, and psychiatric aspects, just like in individuals with SARS-CoV-2 infection [140]. The psychosis clinically presents with panic disorder, post-traumatic stress disorder (PTSD), depression, and obsessive-compulsive disorder (OCD) [141].

## SARS-CoV-2 and endocrine system

### “Endocrine phenotype” of COVID-19

The endocrine system is a significant regulating system as it integrates and controls all the body‘s physiological functions. Any disturbance or defect in the endocrine system may cause mild to severe complications. Viral infection is one of the threatening factors for the endocrine system or one of its glands and functions. COVID-19 endocrine phenotype has gradually gained medical significance [142, 143]. Many endocrine disorders caused by disrupting the hormonal system during and post-SARS-CoV-2 infection can develop into chronic disorders [144]. Numerous endocrine glands, such as the hypothalamus, pituitary, thyroid, pancreas, adrenal and reproductive glands (testes and ovaries) express ACE2 receptors, which is the central binding location of the SARS-CoV-2 virus, as aforementioned [145]. Throughout the crisis of COVID-19, various research articles concentrated on the complications of the endocrine system caused by COVID-19 in endocrine disease patients. Knowing the potential impact of COVID-19 on endocrine glands is essential to clinically control endocrine disturbance before and throughout hospital admission in COVID-19 patients and follow the patient up after improvement [146, 147].

### COVID-19 and the master endocrine gland (the pituitary)

Many studies have discussed the participation of the pituitary gland that suits a gradually forming endocrine phenotype of COVID-19. Furthermore, a particular concern for pituitary disorders is required to continue. However, limits since many pituitary disorders, such as Cushing disease and hypopituitarism, or frequently coupled conditions such as DM might cause a risk factor for acute pituitaries in COVID-19 individuals [148]. A few studies showed disturbance in the pituitary gland in individuals infected with COVID-19. Even though fatality rate from COVID-19 has dropped due to effective vaccinations, numerous studies have focused on the long-term effects of COVID-19 on the endocrine system to investigate appropriate medical care [145, 147]. Patients with pituitary tumors, hormonal irregularities, and a cluster of effects are monitored in multidisciplinary pituitary health facilities because it demonstrates a treatment need in both pandemic and non-pandemic eras. The comorbidities (i.e., hypopituitarism, hypertension, obesity, diabetes, and cardiovascular illnesses) affect the duration and management of COVID-19. As a result, it has been advised that additional attention should be paid to diagnosis, management, and safety guidelines in the COVID-19 pandemic. Individuals with COVID-19 who are older, obese, or have cardiovascular disease and/or diabetes are at a higher risk of hospitalization and mortality. Men will be far more heavily impacted than women, although there is a fair chance that many men, particularly younger ones, will recover.

Furthermore, many pituitary patients are prone to adrenal suppression, hypercortisolemia, Cushing syndrome, adrenal deficit, pseudo diabetes, hypopituitarism, sleep apnea, acromegaly, and chest wall deformity [149]. In ideal settings, endoscopic transsphenoidal pituitary surgery requires complicated orchestration of attention offered by a multidisciplinary professional group of physicians and surgeons’ teams to optimize the three—pre-, intra-, and post-operative procedures. As a result, much tuning is required in the COVID-19 pandemic to play this highly coordinated performance [150]. Pituitary patient care and service must be reconstructed; thus, it is strongly advised to continue using pituitary multidisciplinary treatment clinics employed during and after the COVID-19 crisis [150].

### Effect of COVID-19 on the thyroid gland

COVID-19 can alter thyroid function in various ways, as reported by a couple of published case studies that have indicated the emergence of novel thyroid diseases because of COVID-19. COVID-19 is thought to have an indirect (abnormal systemic inflammatory-immune reactions) and direct effect on thyroid function (viral effect on the gland). Hyperactivity of the Th1/Th17 immune responses, which might contribute to generating and maintaining thyroid gland inflammation, is thought to indirectly affect the thyroid gland [151]. Subacute thyroiditis (SAT) is a thyrotoxicosis-causing inflammatory condition of the thyroid gland that is frequently linked with a viral infection and lymphocytic infiltration. Thyroid pain and tremors, which are not typical of a systemic viral illness, should warrant a thyrotoxicosis study [152].

Thyroid function might be affected both throughout the acute phase of a disease and during the recovery period after COVID-19, even though some data proposes that thyroid dysfunction is triggered by direct infection of the thyroid or an autoimmune effect on the thyroid produced by a cytokine storm [151, 152]. Nevertheless, SARS-CoV-2 has been linked to Graves’ illness in post-COVID-19 cases, for example [153]. Graves’ disease is the most prevalent cause of hyperthyroidism. It is an autoimmune condition that affects the thyroid gland. This affects a 5-10:1 ratio of females to males and peaks in middle age, though it may also affect younger people (children and adolescents) [153]. According to a recent study, individuals with hypo-or hyper-thyroidism are less susceptible to SARS-CoV-2 infection. A diagnosis of hypo- or hyper-thyroidism is not related to an aggravated prediction of SARS-CoV-2 infection when adjusted for comorbidity [154].

Additionally, thyroid disturbance has been reported during COVID-19, such as decreased levels in thyroid stimulating hormone (TSH) and triiodothyronine (T3) along with SAT [156]. It is important to note that ACE2 and TMPRSS2 are abundantly expressed in the thyroid gland, with levels more remarkable than in the lungs. The thyroid and the hypothalamic–pituitary–thyroid axis have been proven as SARS-CoV-2 targets [151]. COVID-19 can be linked to short-term and reversible thyroid abnormalities, although thyroid malfunction is unaffected by COVID-19 [157].

### COVID-19, endocrine and metabolic diseases

According to epidemiological research, older adults with comorbidities have the highest chance of acquiring severe COVID-19 and associated consequences, including mortality. DM, obesity, respiratory, and cardiovascular diseases involving hypertension, and coronary artery disease, are all examples of COVID-19 comorbidities [158]. DM is linked to a higher incidence and severity of COVID-19. High blood glucose has been demonstrated to decrease immunity and increase SARS-CoV-2 replication when combined with other risk factors. In diabetics, oxidative stress and the secretion of pro-inflammatory cytokines are higher than in individuals without DM, exacerbating the result of SARS CoV-2 infection [159].

Diabetes, the most frequent endocrine condition, is a face in the “Endocrine phenotype” for COVID-19 since it is one of the most common comorbidities associated with mortality in COVID-19 patients. Most of the available data focused on glucose dysregulation during COVID-19 infection and the development of diabetes [158, 159]. Thus, individuals infected with SARS-CoV-2 should be given extra attention to the emergence of diabetes.

Obesity, another metabolic, endocrine condition, increases vulnerability to severe SARS-CoV-2 infection. As a result, these patients require proper nutritional balance. Furthermore, vitamin D and Ca+2 deficiencies and fractures were reported as recurring findings in the hospitalized COVID-19 group. Adrenal insufficiency necessitates a change in glucocorticoid medication. Furthermore, sex hormone activities, and odd pituitary and thyroid sides of COVID-19 were found [100]. Following SARS-CoV-2 infection, some individuals developed pancreatitis with elevated amylase and lipase levels [160, 161]. It was recommended to prioritize the treatment of metabolic diseases during and immediately after the COVID-19 pandemic [162].

## SARS-CoV-2 and immune system

Even though COVID-19 is widely known as a viral infection, taking in consideration a variety of scientific papers published so far, one may conclude and define it as a far more complex, heterogeneous, and multi-organ disease—the consequences of which might affect the immune system of an individual [163]. Bearing this in mind, it is essential to highlight those severe forms of COVID-19, which besides causing the viral infection, might also cause the abnormal and aggravated immunological host response that can elicit severe systemic damage [164]. SARS-CoV-2 infection can trigger innate and adaptive immune responses, which in case of being uninhibited or debilitated, could result in harmful tissue damage [165]. However, innate and adaptive immunity participate in the process of viral infection control and the clinical recovery in many infected people [166, 167]. The role of adaptive immunity vital for virus clearance is dysregulated in patients diagnosed with severe COVID-19. In murine models of SARS, MERS, and COVID-19, virus-specific CD4+ T cells, CD8+ T cells, B cells, and antibodies have been found in patients throughout acute sickness and convalescence. They have been demonstrated to be protective and necessary for virus clearance [168–175].

Studies have suggested that during the SARS-CoV-2 infection, the immune response system’s intense activation is often uncontrolled. There is increased anti-inflammatory markers production and interleukins, neutrophils, monocytes, and macrophages. B and T cells for humoral and adaptive immune response, respectively, offer a vital role in the immune response. In response to B cell activation, antibodies are produced. Immunoglobulin M (IgM) is produced initially to provide the initial response. At the same time, immunoglobulin G (IgG) is responsible for long-term immunity and is often detected in serum several days or months after the initial exposure to the virus [176].

Given the evidence of how COVID-19 affects the immune system, its cooperation with other systems, and overall responses, it can be concluded that any disruption of it might cause profound consequences and even lead to the progression of the disease. However, one must bear in mind that to achieve success in both diagnostics and therapeutics, the elaboration of the immune system, its functions, and interactions with other systems must be further studied, better understood, and revealed.

## SARS-CoV-2 and reproductive system

As previously mentioned, SARS-CoV-2 receptors, including ACE2 and TMPRSS2, have been detected in organs of the human body’s numerous systems. Excessive amounts of ACE2 and TMPRSS2 are also seen in the human reproductive system, indicating a possible infection in the primordial germ cells. This cellular invasion disrupts the reproductive glands and may harm the gametes [177]. Men are considered more vulnerable to COVID-19 and have a greater death rate than women [178].

Coronavirus research has revealed that the testes may be a target for SARS-CoV-2 infection. The virus can enter the testes via the ACE2 receptor, according to the initial etiopathogenic idea given by current theories. Following that, an active inflammatory response in the testes, disease-associated fever, and COVID-19 medicines might all be involved in testicular changes [179]. SARS-CoV-2 mRNA and protein were recently found in the sperm of SARS-CoV-2-infected individuals. As a result, the breakdown of the blood–testes barrier (BTB) in febrile disorders is hypothesized during the acute phase of the disease, allowing viral access into the testes. During SARS-CoV-2 infection, spermatogenesis is disrupted—along with the production of gonadotropin (FSH & LH), androgen, and testosterone. Although no sexual transmission has been documented, the virus presence in sperm leaves the possibility of sexual transmission open [180]. Gonad hormones influence immune functions. For instance, estrogenic hormones promote immunity, while testosterone inhibits it. It has been evidenced that the expression of transmembrane protease, serine 2 (TMPRSS2) in lung cells, an enzyme that regulates androgen and expresses essentially in the prostate of adults. This may explain the higher susceptibility of the male to severe COVID-19 than females. ACE-2 works as an active receptor for acute severe respiratory SARS-CoV-2 since male hormones are efficient in the ACE-2 path and facilitate SARS-CoV-2 login in the host’s cells [181]. 5-alpha reductase inhibitors lead to androgen’s signal as the central modulator for ACE2 concentration. Therefore, therapy with the 5-alpha reductase inhibitor, dutasteride, suppresses ACE2 concentrations and reduces the internalization of the R-spike receptor binding domain in some human cell lines. COVID-19 patients showed that disturbance of androgen is markedly connected with severe complications and heart issues as indicated by blood concentrations of troponin T. So, suppression of androgen receptors can act as an excellent therapeutic plan [182].

On the other hand, testosterone has a positive effect on COVID-19 via suppressing pro-inflammatory cytokines, raising those of anti-inflammation, altering immunity response, reducing oxidative stress, and dysfunction of the endothelium. However, its adverse impacts on COVID-19 patients are attributed to an increase in TMPRSS2, the primary enzyme to cleave and activate the S protein of SARS-CoV-2 in acute SARS-CoV-2. While low testosterone concentrations are associated with severe cases of COVID-19 [183]. While some studies revealed that a decrease in testosterone status is a defensive factor, rigorous evidence reported a negative effect between COVID-19 and low gonad activity (hypogonadism) [184].

The viral infection arises (i) testicular damage and inflammatory infiltration. Besides, (ii) viral orchitis due to scrotal pain may manifest, (iii) semen parameters are changed, and (iv) the number of spermatozoa is increased. These findings imply that SARS-CoV-2 infection may cause reproductive difficulties [185]. To perform its physiological duties, ACE2 physically modulates both the expression of angiotensin II and Ang (I-VII). The reproductive system expresses ACE2 abundantly and generates Ang (I-VII), beginning with precursors produced locally or derived from systemic circulation. Ang (I-VII) is an essential stimulant for ovarian follicle development, maturation, and ovulation. Furthermore, the human endometrium expresses Ang (I-VII), mainly during the post-ovulatory phase [186]. Clarifying the effects of SARS-CoV-2 infection on human reproduction would thus give recommendations for women of reproductive age and provide a theoretical basis for the IVF and embryo transfer procedure. Clinical evidence revealed that COVID-19 female infected patients might have ovary disorder represented as a reduction in the ovarian set, anti-Müllerian hormone (AMH), and abnormal amounts of sex hormones such as testosterone and prolactin in the reproductive-endocrine system in a brief period. Therefore, reproductive-aged females should pay extra care to evaluate their ovarian, fertility, and endocrine functions. Recent studies have shown that SARS-CoV-2 infection in pregnant women during pregnancy may affect the newborn‘s hearing. It may relate to intrauterine infection, intrauterine hypoxia, and vertical transmission. Even though the newborn is not an organ of the mother, the newborn and the mother are an integral unit [187]. It has been reported that many factors contribute to ovary dysfunction during and after recovery from COVID-19 (immediate virus offensive, extreme immune response, and hypothalamus–pituitary–ovary axis dysfunction) [186, 188]. Dysregulation of the adrenal gland and spermatogenesis in COVID-19 male patients have also been observed [179, 185].

To infect a cell, SARS-CoV-2 uses two distinct entry pathways: 1) a surface, serine protease-dependent or 2) an endosomal, cysteine protease-dependent pathway [189, 190]. Studies revealed that membrane transmembrane serine protease 2 (TMPRSS2), a protease enzyme responsible for priming S protein from ACE2 receptor, plays an essential role in determining the SARS-CoV-2 entrance pathway [191]. In the TMPRSS2 expressing cells, the virus proteolytic process starts at the host cell’s plasma membrane, and the viral entrance is complete in 10 min. Conversely, when the SARS-CoV-2 interacts with the TMPRSS2-lacking cells, the virus uses the endocytosis pathway, which takes about 40–60 min to complete upon infection [192]. A computational study showed that TMPRSS2 is expressed in various organs, including the lungs, kidney, digestive system, and male tissues [193]. According to the Human Protein Atlas database https://www.proteinatlas.org/, the TMPRSS2 expression is found in the parathyroid gland, lung, salivary gland, stomach, duodenum, small intestine, colon, rectum, pancreas, kidney, and prostate. *In vivo* studies revealed that Tmprss2 knockout significantly inhibits SARS-CoV-2 infection [194]. These results highlighted the importance of TMPRSS2-inhibiting drugs as potential antiviral agents to fight COVID-19, such as aprotinin and SB412515 [192].

## SARS-CoV-2 and skin

The potential of coronavirus to disrupt the overall health picture of an individual has raised concerns even more after the dermatologists’ observations and reports which proved the correlation between cutaneous skin manifestations and patients who have experienced SARS-CoV-2 infection [195–197]. Despite having several patients with symptoms that include cutaneous skin manifestations, the biological pathway that triggers these changes, along with the exact function SARS-CoV-2 has in that process, is still a subject of question [198].

Analyzing the clinicopathological appearances published so far, acral skin symptoms of COVID-19 can be categorized into at least ten distinct types. The following are some of the most common acral skin manifestations of COVID-19: acral papulovesicular eruption, acral urticarial lesion, acral non-inflammatory purpura and necrosis, acro-ischemia associated with COVID-19, acral vasculitis, chilblain-like lesions (COVID Toe), acral erythema multiform (EM), half-moon shaped red bands, and acral peeling [199].

Even though COVID-19-related cutaneous skin symptoms are becoming more common, the pathophysiological mechanisms they exhibit still need to be extensively explored to make as relevant a conclusion as possible.

## SARS-CoV-2 variants and their clinical implications on multi-organ damage

All variants of SARS-CoV-2 arise from infection of COVID-19, but they show differences in the ability of viciousness, diagnosis, and treatment according to different strains of viruses. Variants of concern (VOC) markedly altered the term of COVID-19 epidemiology; owned more viciousness or more transmission ability. SARS-CoV-2 VOC, Omicron (B.1.1.529), may be less severe than Delta (B.1.617.2) or ancestral variants such as Alpha (B.1.1.7), Beta (B.1.351), or Gamma (P.1). Infection symptoms during the Omicron period differ less and significantly from those for the Delta variant. A recent study found that loss of smell, duration of symptoms, involvement of the respiratory tract, and hospitalization rate was less for Omicron [200]. However, a sore throat was more frequent. It should be noted that the symptomatic differences may have resulted from the strengthening of the immune system by vaccination. Additionally, PE was screened during CT pulmonary angiography (CTPA) exams in the three groups of COVID-19 patients infected by ancestral, Delta, or Omicron variants. The incidence of PE in patients infected with ancestral COVID-19 variant, Delta, and Omicron variants were 15.0%, 10.6%, and 9.23%, respectively, reflecting a 41% and 60% decreased risk of PE with Delta and Omicron variants compared to ancestral one [201]. In addition, the viral entry mechanism can differ between Alpha, Delta, and Omicron variants [189], which may affect the prevalence of the disease in different organs and the disease outcome.

## Conclusion

SARS-CoV-2 infection can affect tissues and cells expressing the ACE2 surface receptors in the body, including nerve cells, respiratory, GI, and urinary tract organs. The kidneys are primarily impacted, particularly in the severe type of the illness, which manifests mainly as an AKI. The liver is one of the organs of the GI tract that is considered the second most affected organ by the SARS-CoV-2 infection after the lungs. Additionally, SARS-CoV-2 infection can cause both innate and adaptive immune responses, resulting in harmful tissue damage if uninhibited or debilitated. Since SARS-CoV-2 is an emerging virus with new variants, there is a need to advance scientific research to assess how COVID-19 affects other organ systems and its effect on chronic diseases, such as diabetes, hypertension, and cancer. There is also a need to re-evaluate the existing treatment modalities since the treatment used in SARS-CoV-2 infection can also worsen the disease and to identify better approaches to eradicating the disease and decreasing mortality. Therefore, a holistic perspective is needed in the treatment of the disease. Although this approach is not easily applicable due to the high number of patients, limited sources, and personnel, it will positively affect patients’ recovery in a short time and satisfaction. Although there has been extensive research on COVID-19, there is still minimal current data on some organ systems in different disease stages. This review can help researchers and physicians take a holistic view of the latest developments in multi-organ damage due to SARS-CoV-2 infection.

**Conflicts of interest:** The authors declare no conflicts of interest.

**Funding:** The authors received no specific funding for this work.
